# Supplementary material for: Identification of photoperiod-induced specific miRNAs in the adrenal glands of Sunite sheep (Ovis aries)
Source: Front Vet Sci. 2022 Jul 22;9:888207. doi: 10.3389/fvets.2022.888207 (PMC9354845; doi:10.3389/fvets.2022.888207)
Supplement: Supplementary file 1 [file Data_Sheet_1.ZIP › Supplementary file/miRDeep2 file.zip!/miRDeep2 file/Know_Structure/SPLP42A2/oar-mir-1185.pdf]

miRBase precursor : oar-mir-1185
Total read count : 202
oar-miR-1185-5p read count : 65
oar-miR-1185-3p read count : 137
remaining reads : 0

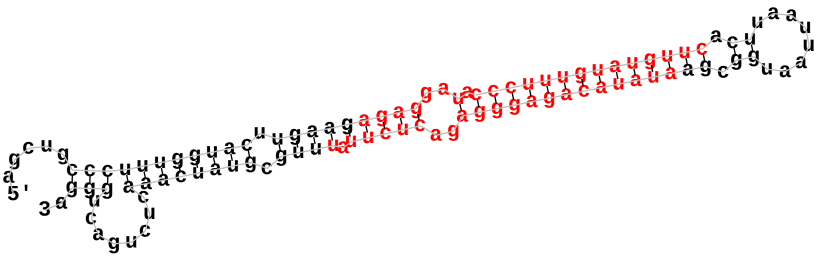

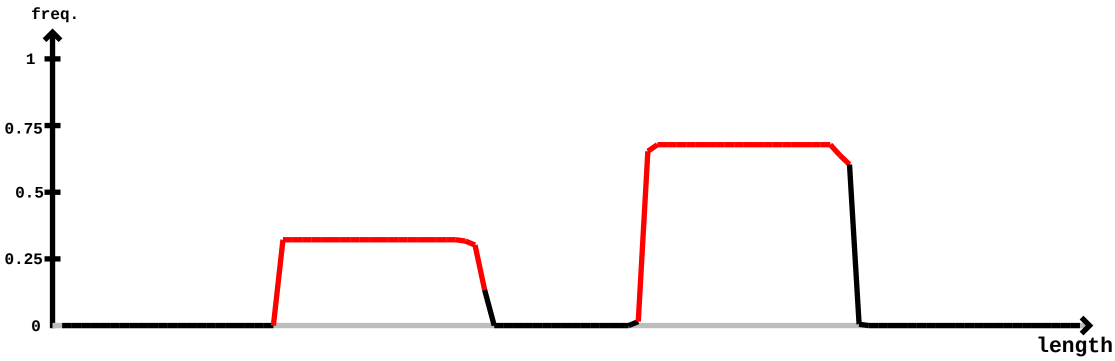

oar-miR-1185-5p

oar-miR-1185-3p

5'- agcugcccuuugguacuugaagagaggauacccuuuguauguucacuuaauuaauggcgaauauacagagggagacucuuauuugcguaucaaacucugacuggga -3'	exp		
.....((((((((((.(((((((((..(.((((((((((((((.((........)).))))))))))))))))..))))).)))).))))))))........))).	reads	mm	sample
......................agaggauacccuuuguaug...	1	0	seq
......................agaggauacccuuuguaugu..	3	0	seq
......................agaggauacccuuuguauguu...	34	0	seq
......................agaggauacccuuuguauguuc..	27	0	seq
....aauauacagagggagacucuuau........................	3	0	seq
....auauacagagggagacucuu..........................	8	0	seq
....auauacagagggagacucuua.........................	7	0	seq
....auauacagagggagacucuuau........................	113	0	seq
....auauacagagggagacucuuauu.......................	1	0	seq
....uauacagagggagacucuuau........................	5	0	seq
